# Metal Sulfides as Sensing Materials for Chemoresistive Gas Sensors

**DOI:** 10.3390/s16030296

**Published:** 2016-02-26

**Authors:** Andrea Gaiardo, Barbara Fabbri, Vincenzo Guidi, Pierluigi Bellutti, Alessio Giberti, Sandro Gherardi, Lia Vanzetti, Cesare Malagù, Giulia Zonta

**Affiliations:** 1 Department of Physics and Earth Science, University of Ferrara, Via Saragat 1/c, Ferrara 44122, Italy; fbbbbr@unife.it (B.F.); ghrsdr@unife.it (S.G.); malagu@fe.infn.it (C.M.); zntgli@unife.it (G.Z.); 2 MNF- Micro Nano Facility, Bruno Kessler Foundation, Via Sommarive 18, Trento 38123, Italy; bellutti@fbk.eu (P.B.); vanzetti@fbk.eu (L.V.); 3 CNR-INO—Istituto Nazionale di Ottica, Largo Enrico Fermi 6, Firenze 50124, Italy; 4 MIST E-R s.c.r.l., Via P. Gobetti 101, Bologna 40129, Italy; giberti@fe.infn.it

**Keywords:** metals sulfides, chemoresistive gas sensors, thick-film, cadmium sulfide, tin (IV) sulfide

## Abstract

This work aims at a broad overview of the results obtained with metal-sulfide materials in the field of chemoresistive gas sensing. Indeed, despite the well-known electrical, optical, structural and morphological features previously described in the literature, metal sulfides present lack of investigation for gas sensing applications, a field in which the metal oxides still maintain a leading role owing to their high sensitivity, low cost, small dimensions and simple integration, in spite of the wide assortment of sensing materials. However, despite their great advantages, metal oxides have shown significant drawbacks, which have led to the search for new materials for gas sensing devices. In this work, Cadmium Sulfide and Tin (IV) Sulfide were investigated as functional materials for thick-film chemoresistive gas-sensors fabrication and they were tested both in thermo- and in photo-activation modes. Furthermore, electrical characterization was carried out in order to verify their gas sensing properties and material stability, by comparing the results obtained with metal sulfides to those obtained by using their metal-oxides counterparts. The results highlighted the possibility to use metal sulfides as a novel class of sensing materials, owing to their selectivity to specific compounds, stability, and the possibility to operate at room temperature.

## 1. Introduction

The great challenge of nanostructured materials lies in the control of their properties by the grain size, which combines bulk and surface effects [1,2,3,4]. Low-dimensional nanostructures have been prepared with various morphologies and have attracted research attention because of their fundamental role in the comprehension of the quantum size effect and great potential applications [5,6]. One-dimensional (1D) nanostructures are ideal for investigating the dependence of electrical transport, mechanical and optical properties on size and dimensionality [7]. Indeed, highly attractive properties and novel applications have resulted from well-aligned one-dimensional nanostructures on substrates, because they play a key role as both interconnections and functional components in improving performance of technologically advanced devices [8,9]. In recent years, many unique and excellent properties have already been demonstrated or proposed, such as superior mechanical toughness, lower turn-on voltage for field emitters, higher efficiency for solar cells, better electrochemical performance for lithium-ion batteries and enhancement of thermoelectric figure of merit [10,11]. At the same time, two-dimensional (2D) nanostructures, *i.e.*, nanosheets, nanoplates, and nanowalls, are suggested to be ideal components for nanoscale devices used in data storage, nanoswitches and biological sensors, due to their nanometre-scale thickness, high surface-to-volume ratio, and fascinating photocatalytic and optical activities [12]. 

In the last years, the variable features of colloidal nanocrystals, such as their size-dependent electronic, optical, magnetic, mechanical and chemical properties, which cannot be obtained in their bulk counterparts, have attracted the attention of researchers [13,14]. Within colloidal semiconductors, metal chalcogenide nanocrystals have been extensively investigated due to their size-dependent photoemission characteristics and quantum confinement effects [15]. These nanomaterials can be used for different biological labelling and diagnostics, electroluminescent devices, lasers, photovoltaic devices, light-emitting diodes and single-electron transistors [16]. Among colloidal nanocrystals, metal oxides have gained a significant role in technology development due to their exceptional skills. In recent times, several research works have been focused on these semiconductors to explore new application fields, such as optical, electronic, optoelectronic and biological domains. In particular, the application in which metal oxides have been widely used is gas sensing. The performance of sensors based on metal oxides depends crucially on their dimensions, morphology, composition and surface activity [17]. Among the several parameters that influence the sensing properties of a metal oxide, the potential barrier at the interface between grains is a major physical quantity [18]. Indeed, in this sense, the broad assortment of one-, two- and three-dimensional metal-oxides nanostructures has been a precious source for gas sensors technology, which owes its constant development to the requirements of physical, chemical and biological detection systems [19,20,21,22]. 

Metal sulfides are nanocrystals with great potential for investigation, due to their various types of structures. They are abundant and cheap because they exist in nature as minerals, *i.e.*, heazlewoodite (Ni_3_S_2_), chalcocite (Cu_2_S), pyrite (FeS_2_) and others. The morphology of metal-sulfide nanostructures can be controlled by applying general solution methods and thermal evaporations, and their possible applications in energy conversion and storage were demonstrated. In the scientific literature, many papers have been reported to provide an overview of recent research and significant advances, ranging from synthesis to properties and applications, especially in energy conversion and storage, such as solar cells, lithium-ion batteries, piezoelectric nanogenerators and fuel-cells [23,24,25]. So far, in the gas sensing field, metal sulfides have been mainly studied in combination with metal oxides in order to modify the sensing activity of the latter [26,27]. Metal sulfides as sensing materials for gas detection have been poorly studied, and the works published do not present an in-depth study about their sensing properties [28,29]. On the contrary, the literature presents extensive investigations on metal-oxide semiconductors as sensing materials, due to their excellent sensitivity, fast response and recovery times, and low-cost [30,31]. However, despite such important advantages, metal-oxides still exhibits unsolved drawbacks. Their incomplete selectivity and lack of stability sometimes result in unreliable responses [32,33]. Moreover, these semiconductors often need a significant amount of energy to support chemical reactions at the surface, activated at high temperatures. By studying physical and chemical properties of nanostructured metal sulfides, it arose that such materials may be very good candidates to be further investigated in the chemoresistive gas sensing field. Indeed, by using these materials, we expect an improvement from an energy consumption point of view, both in thermal- and photo-activation modes, due to their lower band-gap than for metal-oxide semiconductors. This means that the activation of intrinsic surface reactions occurs at lower working temperatures, then minor power supply is necessary. Due to this advantage, we were motivated in the search for potential improved performance in terms of selectivity and stability. The absence of oxygen in the crystal lattice of metal sulfides leads to a different catalytic mechanism on the surface reaction with respect to metal oxides. In addition, this absence may solve the constant drift of the signal suffered by metal oxides and ascribed to the in/out diffusion of oxygen vacancies, which alters the doping level. For these reasons, we decided to focus our work on the use of metal sulfides for chemoresistive gas sensors by means of thick-film deposition technique. 

In this work, the sensing properties of Cadmium Sulfide (CdS) and Tin (IV) Sulfide (SnS_2_) were deeply studied in thermo- and photo-activation mode. Some characteristics of these two metal sulfides, such as the possibility to synthesize them on a nanometric scale with simple and inexpensive methods and their thermal stability, encouraged their usage as sensing films. Moreover, each of these metal sulfides is the counterpart of a metal oxide, in particular, SnS_2_ is analogous to the widely used SnO_2_. Hence, a comparison of the sensing properties and performance between metal sulfides and their metal-oxide counterparts was carried out, in order to identify the main promising features of these semiconductors as gas sensing materials. 

## 2. Experimental Section

### 2.1. Chemical Synthesis and Thick-Films Deposition

Cadmium Sulfide (CdS) and Tin (IV) Sulfide (SnS_2_) were synthesized as nanoparticles via precipitation reactions in aqueous solution, using thioacetamide as a source of S^2−^ ions and metalorganic or salt compounds to release metal ions. The chemical reagents used in these syntheses were from Sigma Aldrich. In order to control the growth of crystals, which was expected to be nanometric, a complex agent was used and chemicals adapted to adjust the solution pH.

#### 2.1.1. Synthesis of Cadmium Sulfide

Cadmium Sulfide nanoparticles were obtained by precipitation method at room temperature and atmospheric pressure, in aqueous solution. In this synthesis, 10 mmol of cadmium acetate dihydrate and 20 mmol of o-phenylenediamine were dissolved in 100 mL of water and stirred for 2 h. Afterwards, 20 mmol of thioacetamide were added to the solution. The mixture obtained was stirred for 6 h. Hence, a precipitate of yellow-orange nanoparticles was formed. The product was isolated by vacuum filtration and washed several times with methanol and water. At last, CdS nanoparticles were dried for 4 h at 40 °C. The synthesis was performed in three different modes: without o-phenylenediamine (sample S1), with 10 mmol (sample S2), and with 20 mmol of o-phenylenediamine as complexing agent (sample S3). 

#### 2.1.2. Synthesis of Tin (IV) Sulfide

The Tin (IV) Sulfide nanoparticles were synthesized through precipitation at standard pressure and temperature, in aqueous solution. First, 1.65 mmol of SnCl_4_·5H_2_O were dissolved in a beaker with HCl (37% m/v). Then, to the resulting suspension were added distilled water, diluted in 80 mL. The solution was stirred for 10 min. Afterwards, 0.25 g of thioacetamide and 20 mL were added to this solution. The mixture obtained was stirred for further 3 h. The Tin (IV) Sulfide precipitated in this solution as brown nanoparticles, thus it was isolated by vacuum filtration and washed with water and methanol. At last, the product was dried for 6 h at 40 °C. Also for SnS_2,_ three synthesis were carried out: without HCl (sample ST1), with HCl as chemical to adjust pH (pH = 3) (sample ST2), and with the same quantity of HCl (pH = 3) and 3.3 mmol of o-phenylenediamine as complexing agent (sample ST3).

**Figure 1:**
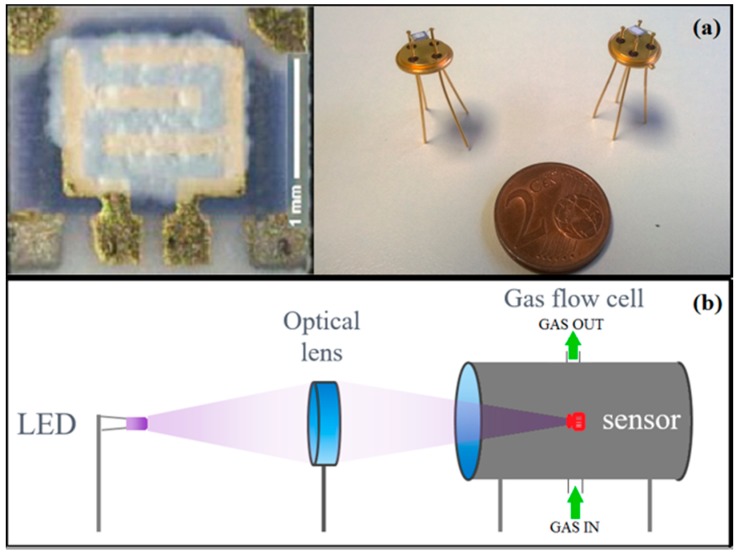
(**a**) Image of the gas sensor device and (**b**) the schematic representation of the gas sensing system used for electrical characterization in photo-activation mode.

Organic vehicles were added to CdS and SnS_2_ nanopowders in order to obtain pastes with a suitable viscosity, to allow the deposition of the sensing layers onto alumina substrates through the screen printing technique (thickness∼30 μm) [34]. In particular, the organic vehicle used was composed of a glycol ether as wetting agent and an acrylic resin. The front-side of alumina substrates provides interdigitated Au electrodes for the measure of the film resistance, while the back-side is equipped with a heater to apply the optimal working temperature of the sensors. Afterwards, to obtain the thermal stabilization, the screen-printed films were treated at 180 °C in a muffle oven for 12 h in air, allowing the evaporation of organic vehicles. The substrates were finally bonded on a suitable support to be connected with the electronic system (Figure 1a).

### 2.2. Chemical, Morphological and Structural Characterizations

Powders and films were studied with Energy Dispersive X-Ray spectroscopy and Scanning Electron Microscopy (SEM-EDX spectroscopy) techniques, to investigate morphology and chemical composition of the obtained materials. The instrument used was a Zeiss EVO 40 Microscope with an acceleration voltage of 30 kV.

Further information on powders were by TEM images, obtained by a Hitachi H800 microscope, supplied with a tungsten gun with maximum voltage of 200 kV. 

X-Ray Diffraction (XRD) analysis was carried out on the as-synthesized CdS and SnS_2_ nanopowders and on the thermal processed powders. The instrument was a Bruker D8 Advance diffractometer equipped with a Si(Li) solid-state detector(SOL-X) set to measure CuKα 1,2 radiation and with an X-ray tube operating at 40 kV and 40 mA. An alumina and zero background holders were used as side loaded for the samples, respectively. Measuring conditions were 5–95° 2θ range, 0.02° 2θ scan rate, counting time per step 4 and 6 s for as synthesized and thermally treated nanopowders, respectively. The phase identification was achieved by search-match using the EVA v.14.0 program by Bruker and the Powder Diffraction File database (PDF) v. 9.0.133. To define the crystallite size of the nanopowders, the TOPAS v.4.1 program was used, based on the Rietveld method and accomplished by the Double–Voigt approach [35,36]. The line-profile fitting was obtained through the fundamental parameters [37,38,39].

For XPS measurements, the powders were attached to the sample holder using a double-sided carbon tape. XPS spectra were recorded using a Scienta Esca-200 system equipped with a monochromatized Al Ka (1486.6 eV) source. An overall energy resolution of 0.4 eV was routinely used. The emission angle between the axis of the analyzer and the normal to the sample surface was negligible. For each sample Sn 3d, S 2p, O 1s and C 1s core levels were collected. Charge compensation was achieved using a flood gun and all core level peak energies were referenced to the saturated hydrocarbon in C 1 s at 285.0 eV.

The thermogravimetric analysis (TG/DTG/DTA) of the sensing materials were carried out using a Netzsch 409 PC Luxx TG/DTA thermal analyzer. A proper amount of samples were filled in a nickel crucible and analyzed in the range 20–800 °C, with a heating rate of 10 °C∙min^−1^ under air flow of 20 mL∙h^−1^.

UV–vis absorption measurements were performed to investigate the optoelectronic properties of the synthesized semiconductor, by using a Cary 50 Varion instrument in the range 300–900 nm (Virtual double-radius). The dimethyl sulfoxide was used as reference and solvent. The cuvette was made of quartz and the optical path was 1 cm.

### 2.3. Gas Sensing Measurements

SnS_2_ and CdS sensors were electrically characterized in a dedicated chamber for gas measurements by means of the flow-through technique. The sensors were heated at their working temperature under a continuous flow of synthetic air for a few hours before testing the gases, in order to achieve the thermodynamic equilibrium of the SnS_2_ and CdS grains on the surface. Air and gases were from certified bottles and their injection in the chamber was carried out by means of a PC-driven mass-flow-controller. The conductance of the films was constantly recorded during the gas measurements through proper electronics interfaced to a data-acquiring system. 

For a *n*-type semiconductor, the responses to reducing and oxidizing agents were calculated as:

(1) $$=\{\begin{array}{c} \left(G_{gas}-G_{air}\right)/G_{air}\;for\;reducing\;gases\\ \left(G_{gas}-G_{air}\right)/G_{gas}\;for\;oxidizing\;gases \end{array}$$

where *G_gas_* and *G_air_* are the conductance values in gas and in air, respectively.

#### 2.3.1. Gas Measurements in Thermo-Activation Mode

The performance of the sensing films was investigated at operating temperatures ranging between 150 °C and 300 °C for SnS_2_-based sensors, and between 150 °C and 350 °C for CdS-based sensors. Higher temperatures must be avoided with these materials because they oxidize at temperatures of 400 °C and 500 °C for SnS_2_ and CdS, respectively [40,41,42]. For this reason, we decided to test these sensors at temperature fairly lower than their critical temperature. Both the sensors highlighted, at temperatures lower than 250 °C, an extremely low chemoresistive activity. This result proved that temperatures lower than 250 °C are not sufficient to activate chemical reactions at the surface. 

The tested gases represent different categories of molecules, and, in this way, it was possible to verify the surface reactivity of these semiconductors with respect to analytes characterized by important chemical differences. Gas concentrations were chosen taking into account the corresponding Threshold Limit Value (TLV) [43].

#### 2.3.2. Arrhenius Plot and Intergrain Barrier Measurements 

Arrhenius plot and intergrain barrier *vs.* temperature measurements were performed to compare the behavior of metal sulfides with their corresponding metal oxides. The analysis was conducted on the thick films at temperatures ranging within 100–500 °C. All measurements were carried out in a sealed chamber at 25 °C under atmosphere controlled with a constant flow rate (0.5 L/min) of synthetic air [44].

#### 2.3.3. Gas Measurements in Photo-Activation Mode

For the electrical characterization in photo-activation mode, the sensors were placed in a dedicated chamber provided with a glass window, through which the light emitted by a Light Emitting Diode (LED) was focused onto the films by means of an optical system (Figure 1b). The LEDs were quasi-monochromatic with an emission spectrum width of 5 nm. Synthetic air and gases were injected into the chamber by the flow-through technique. The electrical conductance of the films was recorded continuously during the experiments by using a simple circuit based on an operational amplifier. The films were maintained under a continuous flow of synthetic dry air during all experiments. At first, CdS and SnS_2_ sensors were tested in dark condition. In this case, we observed that the conductance was too low to be measured with our experimental setup (<10^−1^ °·Ω^−1^). In order to achieve an overview about chemoresistive properties of the two photo-activated metal sulfides, we decided to use different excitation wavelengths ranging from 400 to 645 nm. For each incident radiation wavelength, we measured the conductance variation of the films during the injection in the chamber of controlled concentrations of CO (10 ppm), H_2_S (10 ppm), benzene (2 ppm), ethanol (10 ppm), and methane (2500 ppm). Selected concentrations were based on the human exposure limits [43]. 

## 3. Results and Discussion

### 3.1. Powders and Films Characterizations

The preparation of pure nanostructured powders is crucial to obtaining high performance thick films. It is very important to obtain regular and nanoscale structures, since the large number of atoms on the surface and the effective Van der Waals, Coulombic and interatomic coupling significantly modifies the physical and chemical properties of low dimensional materials. Nanostructures characterized by a small size and high surface-to-volume ratio are the best candidates to improve the capability of detecting chemical and biological species [45]. For this reason, a deepened characterization of the obtained products is fundamental to improving the synthesis process for achieving the best possible sensing material.

#### 3.1.1. Cadmium Sulfide Characterizations

**Figure 2:**
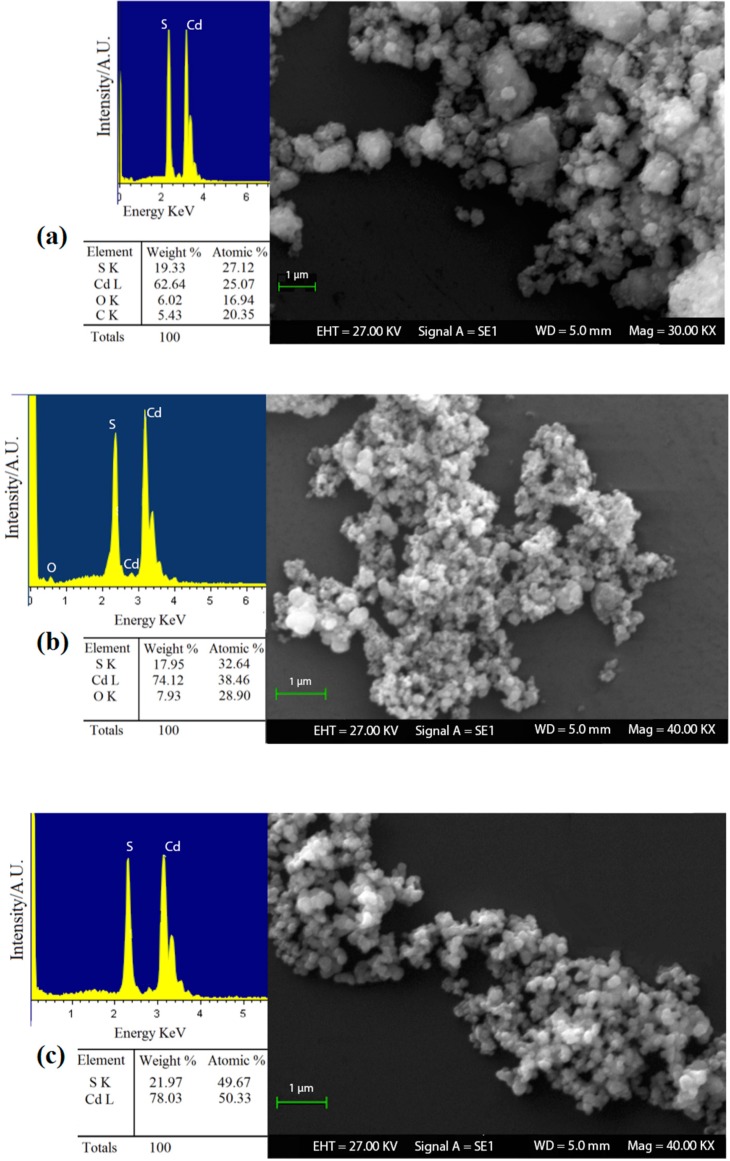
SEM-EDX analysis on CdS powders of (**a**) sample S1; (**b**) sample S2; and (**c**) sample S3.

In order to investigate the role of o-phenylenediamine and the advantages that could lead to the formation of nanostructured cadmium sulfide, it was decided to analyze the different products obtained (S1, S2 and S3) with SEM-EDX analysis. The results highlighted that the reaction methods, in which o-phenylenediamine was used, allows obtaining a product with a higher chemical purity and reaction yield (Figure 2). In fact, EDX analysis on sample S1 (Figure 2a) showed a high quantity of carbon and oxygen, in addition with an average size of the crystal grains (≈400 nm) greater than samples S2 and S3 (Figure 2b,c, with an average size of ≈200 nm and ≈100 nm, respectively). These results highlighted the important role of o-phenylenediamine as a complex agent. In fact, the chemical reaction was expected to occur as a two-stage process:

Cd^2+^ + 2o-phenylenediamine → [Cd(o-phenylenediamine)_2_]^2+^ (2)

In this step, cadmium acetate was dissolved in water to give the Cd^2+^ ions. O-phenylenediamine reacts at this time with Cd^2+^ ions, resulting in the formation of a metal–ligand complex. The thioacetamide was ready decomposed and acted as the sulfur source generating S^2−^ that allowed the formation of CdS nanoparticles

[Cd(o-phenylenediamine)_2_]^2+^ + S^2−^ → CdS + 2o-phenylenediamine (3)

The SEM-EDX results showed the importance of intermediate metal–ligand complex formation to nanocrystal arrested precipitation growth kinetics, particle stabilization, and, ultimately, their optical properties [46,47]. At the same time, in Figure 2, the difference between samples S2 and S3 can be noted. The latter exhibits a greater chemical purity, ideal stoichiometry and lower grain dimensions than the former. In addition, the reaction yield for sample S3 (98, 5%) was better than sample S2, with which 80% of the product was obtained compared to the theoretical value achievable. These data support the idea that specific cadmium complex formation modulates the crystal growth. The comparison between samples S2 and S3 has also provided further evidence to support the previous literature [48], in which it is suggested that o-phenylenediamine acts as a bidentate ligand with Cd^2+^ ions leading to the formation of a tetrahedral or square planar coordination complex. Therefore, in sample S2 o-phenylenediamine behaves as a limiting reagent with respect to cadmium ions, and it failed to coordinate completely Cd^2+^ ions [49]. From the obtained results, we decided to use the powder S3 as sensing material and to perform further characterizations on this sample only. 

**Figure 3:**
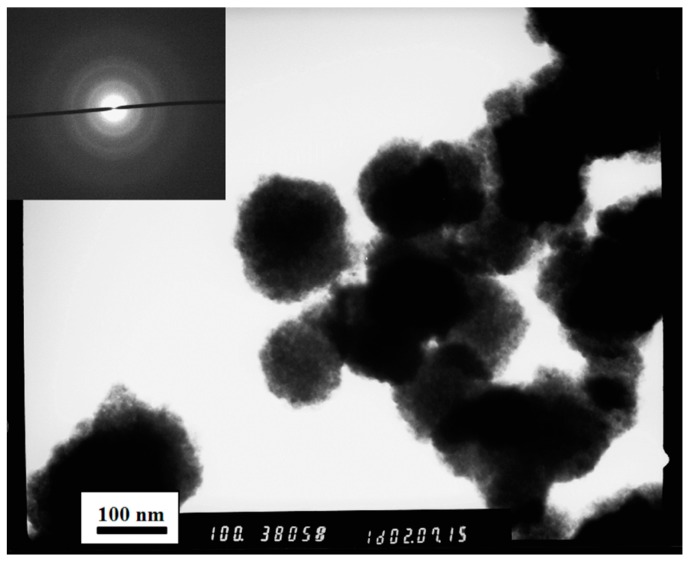
The TEM image of the CdS nanopowder, sample S3. The inset shows the SAED diffraction pattern of the CdS sample.

The additional characterizations, as reported in [42], allowed for a very high chemical purity of CdS to be obtained. The XRD analysis showed monophasic material and the peaks correspond to the cubic polymorph of Cadmium Sulfide (space group F−43 m, corresponds to β-CdS / hawleyite). The crystallite size resulted 2.59 ± 0.14 nm. TEM analysis, reported in Figure 3, shows that the clusters previously observed with SEM analysis are composed by structures with nanobead-like morphology. The average size of clusters was about 100 nm, while the dimension of nanobeads is roughly a few tens of nanometers. SAED diffraction pattern is shown as an inset in Figure 3. It was possible to calculate the interplanar distances of crystallites, knowing the wavelength of the electrons used for the analysis, the camera length of the TEM and the radius of each diffraction ring. The interplanar distances were 3.2, 2.1 and 1.7 Å, which confirmed XRD analysis results. Thermal analysis highlighted that no significant change occurs in the material up to about 500 °C, and this suggests a stoichiometric and morphological stability. In UV-visible analysis, an absorption peak blue-shifted can be noted compared to the characteristic peak at 515 nm of bulk cubic Cadmium (II) Sulfide, due to the electronic transitions. This phenomenon is known as “quantum size effect” [50,51].

#### 3.1.2. Tin (IV) Sulfide Characterizations

**Figure 4:**
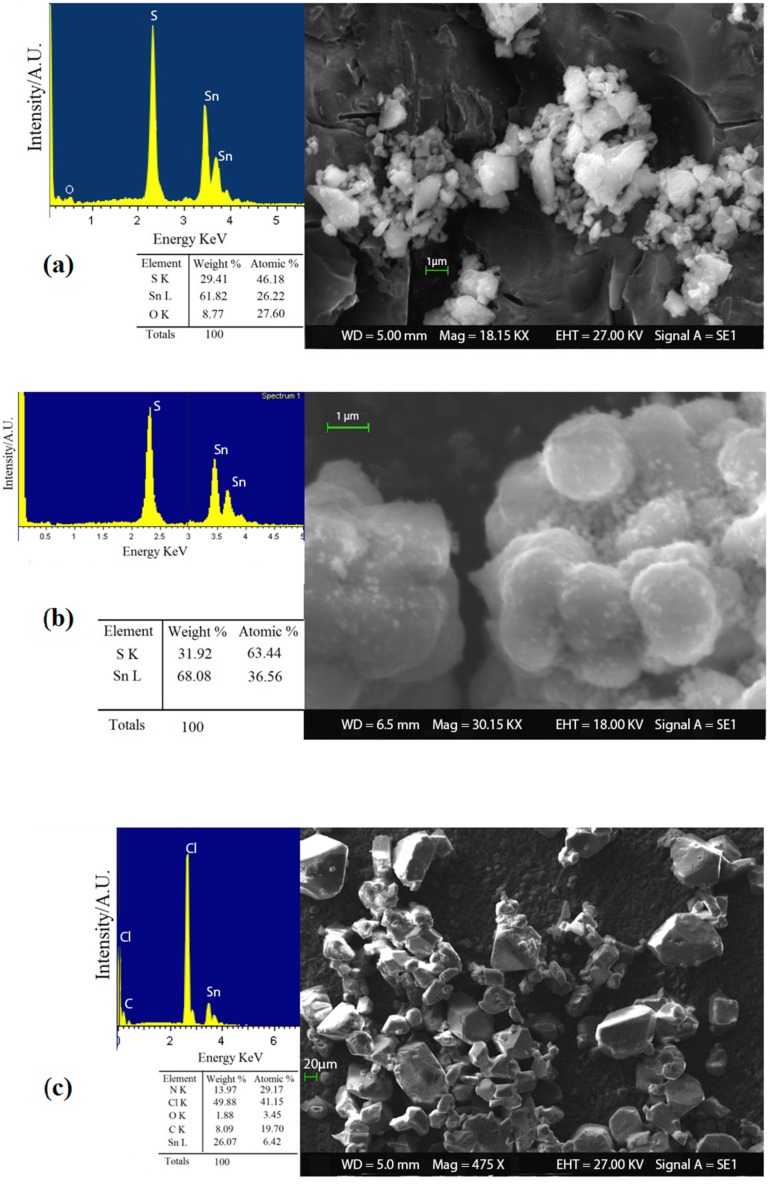
SEM-EDX analysis on SnS_2_ powders of (**a**) sample ST1; (**b**) sample ST2; and (**c**) sample ST3.

In order to investigate the effect of o-phenylenediamine and the acid catalysis on the reaction method to synthesize SnS_2_ nanopowders, it was decided to analyze the different products obtained (ST1, ST2 and ST3) with a preliminary SEM-EDX analysis. As can be seen in Figure 4, the use of o-phenylenediamine and the subsequent formation of the complex in water solution with Sn^4+^ ions did not lead to a real advantage for the synthesis route. In fact, the sample ST3 (Figure 4c) obtained with the complex agents results in the formation of very large (≈ 10 µm) and impure grains that contain high levels of by-products identified with EDX chemical analysis, including compounds with chloride, nitrogen, oxygen and carbon. Conversely, the acid catalysis played a key role in the formation of pure and nanostructured SnS_2_. Indeed, product obtained in acidic solution (ST2) achieved a better reaction yield (95%) than sample ST1 (83%). Moreover, SEM-EDX characterization in Figure 4b showed that sample ST2 exhibits a higher chemical purity and smaller nanostructured grains than ST1, Figure 4a (average size of ≈ 300 nm and ≈1 µm, respectively). The role of acid in the synthesis could be attributed to the shift of the chemical equilibrium in the formation of S^2−^ ions. In fact, thioacetamide reacts with the water solution to form hydrogen sulfide [52,53]:

CH_3_C(=S)NH_2_ + H_2_O → CH_3_C(=O)NH_2_ + H_2_S (4)

The dissociation reaction of weak acid H_2_S is one that provides at this moment ions S^2−^ to the solution:

H_2_S ⇌ H^+^ + HS^−^ ⇌ 2H^+^ + S^2−^ (5)

The addition of hydrochloric acid shifted the equilibrium of the reaction towards the products according to the Le Chatelier principle. Consequently, the last reduces the H_2_S dissociation and, hence, the S^2−^ available concentration. The slow release of sulfide ions thereby promoted the controlled growth of the SnS_2_ grains.

From the results obtained, it was decided to use the ST2 powder as sensing material and to perform the others characterizations only on this sample. 

**Figure 5:**
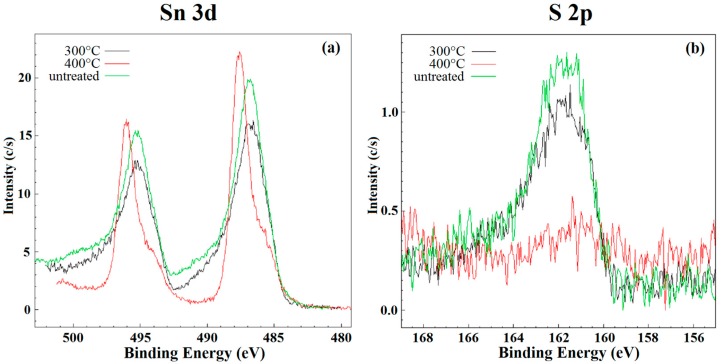
XPS analysis on SnS_2_ powder untreated (green line), after heat treatment at 300 °C (black line) and after heat treatment at 400 °C (red line) for (**a**) Sn 3d and (**b**) S 2p.

The additional characterizations, as reported in [40,41], allowed obtaining a very high chemical purity of SnS_2_. The clusters observed in SEM images are composed by structures with nanorod-like morphology. The average dimensions of nanorods were about 20–30 nm length and 5 nm thick. Indeed, the material became monophasic (space group P-3m1, corresponds to Berndtite-2T), and the crystallite size was 6.18 ± 0.42 nm. The thermal analysis highlighted the stability of as-synthesized SnS_2_ nanopowder at temperatures above 350 °C. This stability was confirmed, in Figure 5, by the XPS results. In fact, the line shape and peak position of Sn 3d and S 2p core levels (typical of SnS_2_) did not change for the samples before and after the heat treatment at 300 °C for five days. On the contrary, these peaks changed with the treatment at 400 °C for five days. The shift of tin peaks is due to the fact that the transition of SnS_2_ to SnO_2_ occurs at this temperature. At the same time, the peak of sulfur almost disappeared confirming the ongoing chemical transitions to SnO_2_.

### 3.2. Gas Sensing Characterizations

#### 3.2.1. Gas Sensing in Thermo-Activation Mode

**Figure 6:**
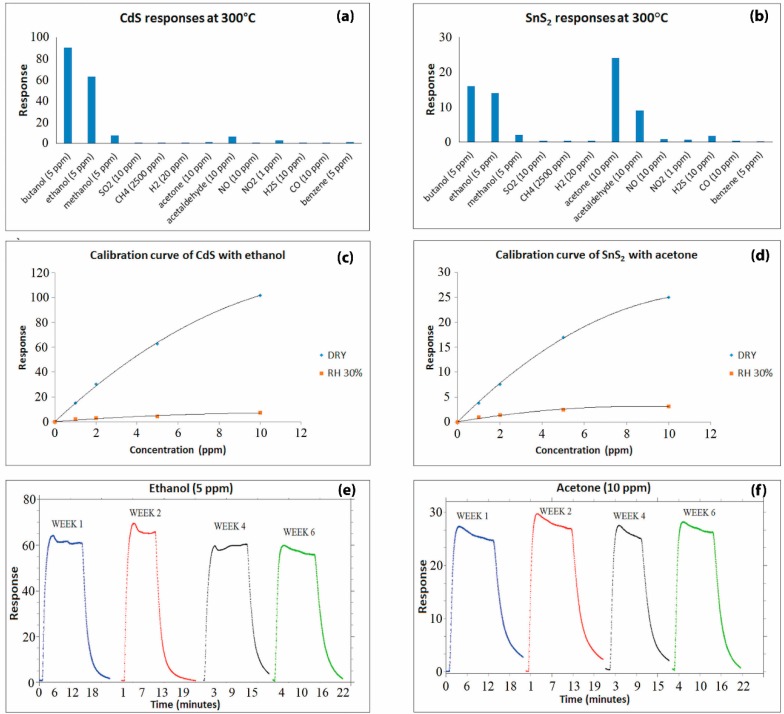
Gas measurement results obtained in thermo-activation mode: (**a**) and (**b**) Selectivity; (**c**) and (**d**) Sensitivity; (**e**) and (**f**) Stability of Cadmium Sulfide and Tin (IV) Sulfide, respectively.

The approach we followed to investigate the gas sensing properties of CdS and SnS_2_ thick films ensued the ”3S-rule”, *i.e.*, sensitivity, selectivity and stability. Through the temperature spectra, it was found that the best chemoresistive behavior of CdS and SnS_2_ films occurred at 300 °C. At this temperature, the layers exhibited high selectivity to specific chemical groups, as can be seen in Figure 6. Electrical characterization covered a broad selection of gaseous compounds. CdS-based sensors were selective to alcoholic groups (Figure 6a), as reported in [42], whereas SnS_2_ layers were capable of discriminating between the carbonyl groups of aldehydes and ketones, as reported in [40,41], as well as the hydroxyl group of the alcohols (Figure 6b). The response time as well as the recovery time was in the order of a few minutes for both sensing materials, similar to those of traditional metal oxides [54]. 

Film sensitivity was first studied under dry air conditions, using acetone with SnS_2_ and ethanol with CdS as sample gases. Figure 6c,d shows the calibration curves of CdS and SnS_2_ responses *vs.* gas concentration, respectively. It is well known that the slope of this curve represents the sensitivity of a gas sensor. As can be seen in these figures, the trend demonstrated in the calibration curve is in line with the trend of the common metal-oxide gas sensors [55,56]. Furthermore, the sensing behavior in wet air conditions was investigated. Humidity was injected in the test chamber fluxing dry air in a bubbler containing water [57]. Relative humidity was controlled with a commercial HIH-4000 humidity sensor (accuracy ± 3.5%). A decrease in the gas sensors’ response by more than one order of magnitude was recorded for both CdS and SnS_2_ films. This effect is due to a competitive interaction between the analyte and OH^−^ group on the sensing films. It was verified that humidity levels greater than RH% = 20%–30% had no significant effects on the conductance of the sample. Moreover, it is important to consider that the reduction factor was almost independent of the gas tested, and then the presence of water vapor diminishes the sensitivity but does not alter the selectivity obtained in dry air [41]. Owing to the high selectivity of the two metal-sulfide gas sensors and their behavior under humid conditions, it can be inferred that the combination of the two sensors can be used for simultaneous detection of aldehydes and ketones, as well as alcoholic compounds.

In order to prove the material stability, the responses of CdS and SnS_2_ thick films were analyzed over time, with 5 ppm of ethanol and 10 ppm of acetone, respectively. The results reported in Figure 6e,f highlight a reproducible behavior during this time, a period of six weeks. The main features observed are a very modest response variation and the absence of a definite trend.

A comparison of the chemoresistive characteristics of the metal sulfides with their metal-oxide counterparts (CdO and SnO_2_) was undertaken. Indeed, it is well known that, among the metal oxides widely studied, SnO_2_ is deeply employed for gas sensing devices, thanks to its great chemical and physical properties [58,59]. Instead, CdO was rarely used in this field. In order to obtain comparable measurements, the grains of CdO and SnO_2_ nanostructures used for the comparison had the same size and shape to those of CdS and SnS_2_ grains, respectively. The thickness of each films obtained through screen printing was∼30 µm.

**Figure 7:**
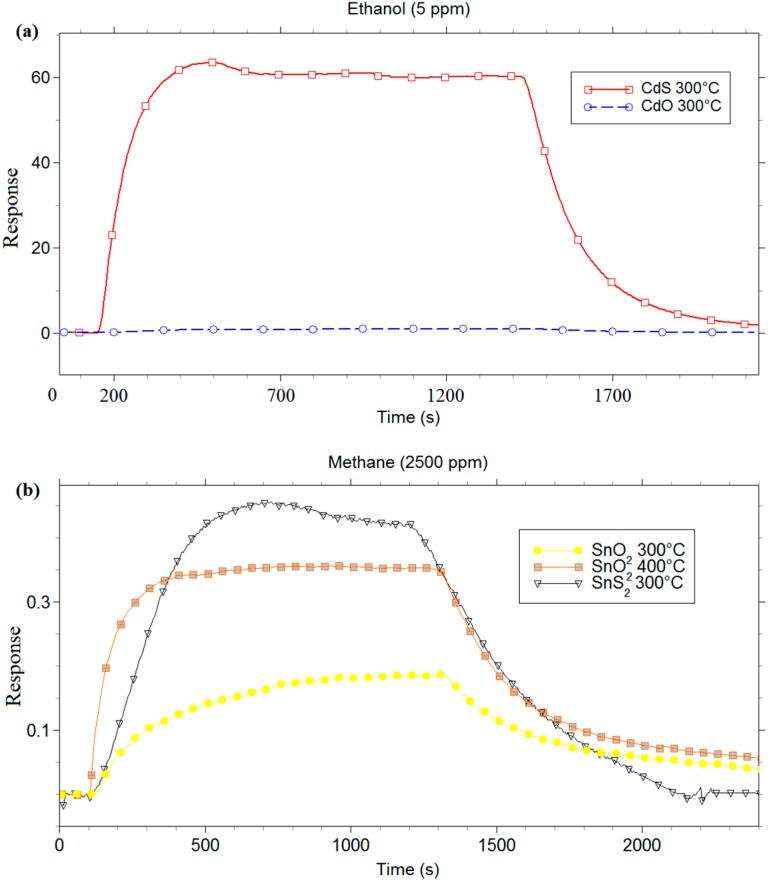
Response in thermo-activation mode of (**a**) CdS and CdO to 5 ppm of ethanol; and (**b**) SnS_2_ and SnO_2_ to 2500 ppm of methane.

Firstly, an electrical characterization of metal-oxide and metal-sulfide films *vs.* gases in dry condition was carried out. Figure 7 shows the results obtained with 5 ppm of ethanol for CdS and CdO, and with 2500 ppm of methane for SnS_2_ and SnO_2_. Several measures were carried out to identify the best working temperature of all films *vs.* tested gases. The comparison between CdO and CdS (Figure 7a), carried out at the relative best working temperature (300 °C) for both sensing materials, highlights the negligible response of CdO with respect to CdS. The comparison between SnS_2_ and SnO_2_ (Figure 7b) was realized in two ways: first, the sensing responses of two layers were compared at 300 °C, which is the best working temperature for the SnS_2_ films. Figure 7b shows that the response of SnS_2_ sensor was higher than the SnO_2_ one, and the response/recovery times of SnS_2_ were faster than SnO_2_. However, 300 °C is not the best working temperature to detect methane for the SnO_2_ sensor. In order to obtain a comparison between SnS_2_ and SnO_2_ sensors at their best working temperature, the measure was also performed by using SnO_2_ sensor at 400 °C. SnS_2_ and SnO_2_ sensors showed a similar trend at the best working temperature, with a slightly higher response and faster recovery times for SnS_2_, as it can be seen in Figure 7b. 

The measurements carried out up to here highlighted the great sensitivity and stability of the two metal-sulfide films. In addition, the better chemoresistive behaviour of CdS than CdO and the excellent sensing properties of SnS_2_ at working temperatures lower than that of SnO_2_ have been observed.

**Figure 8:**
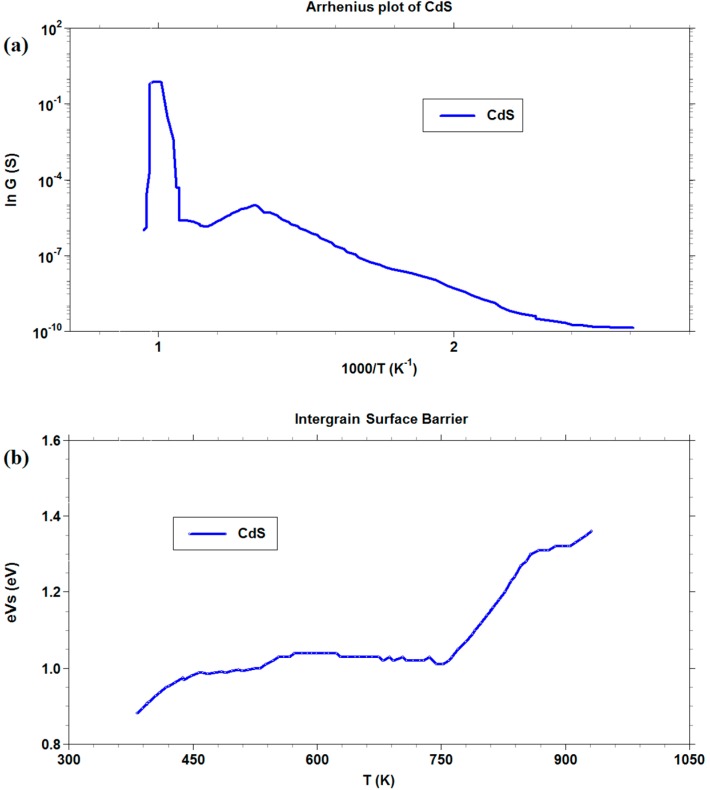
Arrhenius plot and intergrain barrier measurements for CdS thick film.

We decided to further investigate only the CdS films, whereas CdO layers exhibited too poor chemoresistive properties with the gas tested. In Figure 8, both in Arrhenius plot and in barrier measurement, a drastic change in slope was observed at the temperature of about 750 K. A linear increase of barrier with a temperature increase was recorded above this value. We assumed that the change was mainly attributable to the transition of the CdS to CdO, as confirmed by thermal analysis. The rapid increase in the barrier height, once the transition occurred, reflected the decrease in conductance observed in the graph from 740 K to 900 K. A possible cause for this trend may be the oxygen-chemisorbed species at surface, whose reactivity increases after the material has almost completely turned from CdS to CdO, resulting in larger surface negative charge, responsible for a sharp barrier increase [60].

**Figure 9:**
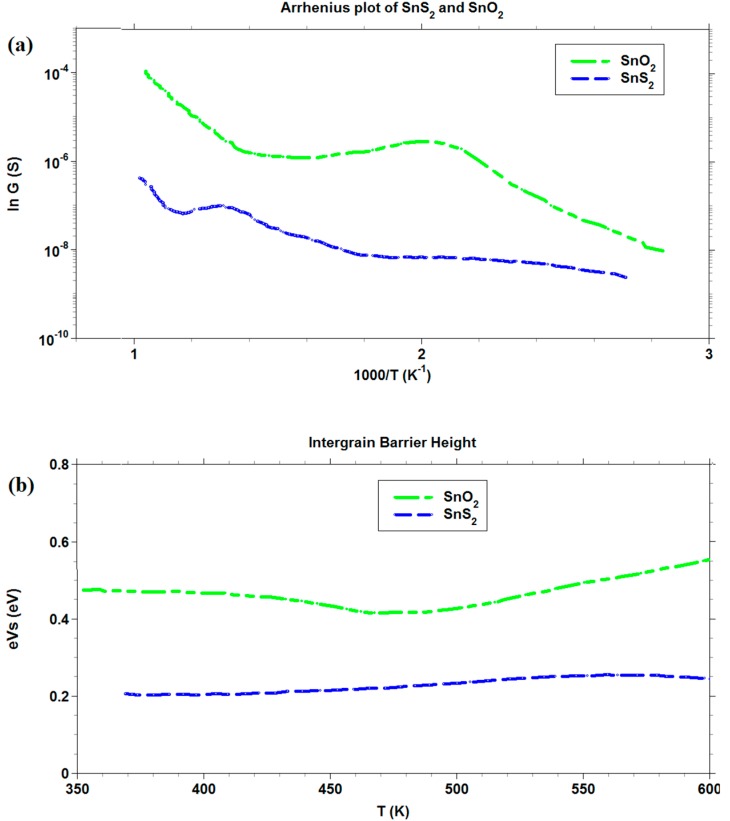
Arrhenius plot and intergrain barrier measurements for SnO_2_ and SnS_2_ thick films.

Regarding the barrier height of SnS_2_ and SnO_2_ films, Figure 9b, a very weak dependence on temperature was observed for SnS_2_ with respect to SnO_2_. The widely acknowledged behavior of SnO_2_, in the range of temperatures chosen [60], showed a net change of slope at temperatures (about 450 K) suitable for the transition of O^2−^ chemisorbed species to O^−^ (which is expected to be more reactive in electron extraction), which was not present in the SnS_2_ barrier curve. This feature explained the change in the slope above 450 K. However, the effect should not be present in SnS_2,_ and its intergrain barrier should remain constant. From the comparison of the Arrhenius plots of SnO_2_ and SnS_2_, reported in Figure 9a, it arose that the conductance *G* of the latter was always lower than for the former. However, the functional dependence of *G* on temperature *T* was more similar at higher temperatures than at lower temperatures. In the temperature region of transition from SnS_2_ to SnO_2_ (about 670 K), a spike in conductance for SnS_2_ appeared, ending in a similar slope to that of SnO_2_ above 800 K. This was a confirmation of the transition of SnS_2_ to SnO_2_, according to TG analysis.

**Figure 10:**
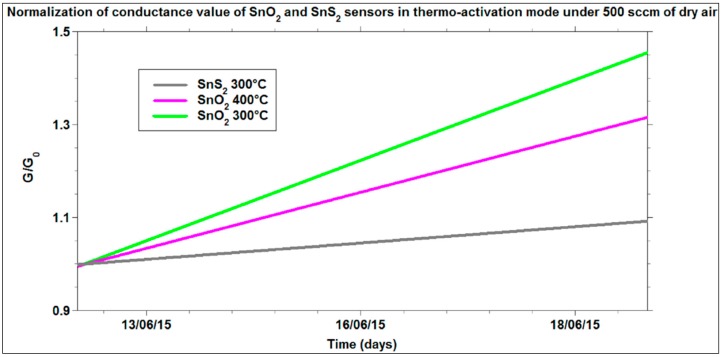
Normalization of the conductance variations of SnO_2_ and SnS_2_ under 500 of dry air (20% O_2_ and 80% N_2_) over the time.

Figure 10 shows another interesting comparison about the conductance values measured for SnS_2_ and SnO_2_ films, exposed to dry air flow over a relatively long acquisition time. All the sensors tested were used for the first time. The main characteristic that can be observed is the drift of the signals for all the films, which represents the initial stabilization trend of these sensors over the first 10 days of use. However, the sensors based on SnS_2_, tested at the best working temperature, highlighted a lower drift of the signal than for SnO_2_ at its best working temperature (400 °C). This superior signal stability of SnS_2_ cannot be ascribed to the different operational temperatures. In fact, by repeating the measurements at lower temperature for SnO_2_ (300 °C), the drift of the signal was always larger than for SnS_2_ at 300 °C. This difference of signal drifts could be explained through the in-out diffusion rate of oxygen vacancies that changes the doping level [61]. In the case of SnS_2_, instead, the cause of *n*-type doping is still under investigation, however, until oxygen is not present in the lattice (temperatures lower than 670 K), the influence of partial oxygen pressure on the signal is expected to be weaker.

In recent literature, other researchers have investigated the possible use of SnS_2_ and CdS as gas sensors. Ou *et al.* [62] have demonstrated the sensitivity of SnS_2_ films to NO_2_, by exploring the sensing behavior of this material with five target gases (H_2_, H_2_S, CH_4_, NO_2_ and CO_2_), whereas Shi *et al.* [28] prepared SnS_2_ nanostructure through hydrothermal synthesis and they studied its sensing behavior to ammonia at room temperature. A brief comparison between responses obtained at room temperature with SnO_2_, SnS_2_ and SnS_2_-SnO_2_ hybrid materials was carried out *vs.* ammonia by Xu *et al.* [27], but the grains of SnO_2_ and SnS_2_ which composed the films had different morphological features. Regards CdS films, Fu *et al.* [29] synthetized this material with leaf-like morphology through hydrothermal method and studied its sensing properties exposed to four gases (isopropanol, methanol, acetone and ether) in thermo-activation mode. Other works on the use of CdS as gas sensors are present in the literature, but in these studies the transduction of the signal depends on catoluminescence [63], Field Electron Transistor [64] and piezoelectric effects [65], and not on chemoresistive mechanisms. 

In the study here presented, which is an extension of preliminary works on the sensing properties of metal sulfides [40,41,42], CdS and SnS_2_ were tested with 13 gases, which belong to different chemical classes, in thermo-activation mode. The behavior was investigated in dry and humidity conditions. The materials showed high selectivity to carbonyl (aldehydes and ketones compounds) and hydroxyl group (alcoholic compounds). Furthermore, the stability of sensors was demonstrated over time; thus, interesting sensing properties were observed in comparing CdS and SnS_2_ films with their metal-oxide counterparts (CdO and SnO_2_). Hence, the results obtained and discussed represent a deeper insight into the sensing properties of these structures, with respect to the state of the art.

#### 3.2.2. Gas Sensing in Photo-Activation Mode 

Concerning SnS_2_ thick films, the results obtained in photo-activation mode did not show a significant change of conductivity. Therefore, SnS_2_ layers are not suitable as photo-activated chemoresistive gas sensors with light sources having wavelengths ranging from 400 to 645 nm. 

Regarding CdS, the results obtained, published in [66], showed the interesting behavior of this semiconductor in photo-activation mode. Indeed, CdS films showed under light irradiation at room temperature a fast and reversible response to gases tested. Furthermore, an important result consists of the observation of different surface chemical activity used with different wavelengths of excitation on nanostructured CdS films. In fact, band gap-resonant excitation turned out to maximize not only the photoconductivity, as found in early works about CdS [66], but also the surface chemical activity. This behavior has been observed for all tested gases, and so it could be expected that it is an intrinsic feature of CdS in photo-activation mode. The observed properties are very interesting concerning the use of CdS as low consumption gas sensors [67].

## 4. Conclusions

This work presented an investigation of the usage of metal sulfides for chemoresistive gas sensing, with particular emphasis on two materials, *i.e.*, Cadmium Sulfide and Tin (IV) sulfide. A preliminary study allowed identifying the best chemical process to obtain the nanostructured powders for both semiconductors. The powders were used as functional materials to obtain thick films, printed through a screen-printing technique. The obtained devices were electrically characterized under thermo- and photo-activation modes.

The layers were verified through the “3S-rule” in thermo-activation mode. SnS_2_ and CdS showed a high selectivity and a sensitivity comparable to the films used for gas sensing based on metal oxides. We also investigated the stability of SnS_2_ and of CdS gas sensors, which highlighted a repeatability of the performances over six weeks with very satisfying results. Moreover, in order to further study the gas-sensing properties of these metal sulfides, comparison measurements were carried out with their metal-oxides counterparts. Among the results obtained, CdS showed better chemoresistive properties than CdO, while SnS_2_ presented a lower signal drift than SnO_2_. 

In photo-activation mode, CdS thick films showed an interesting photochemical activity, which allows for possible use at room temperature and low consumption gas sensing.

It would be interesting to further study metal sulfides in the gas-sensing field in terms of the synthesis and electrical characterizations of different dimensional structures. In particular, 2-D nanostructures, which showed a better stability for metal oxides than 1- and 3-D nanostructures, could allow for interesting future developments. 

Based on the strength of their gas responses, as shown in this paper, one can conclude that metal sulfides such as SnS_2_ and CdS are suitable materials for chemoresistive sensors.
